# Deep Learning-Based Next-Generation Waveform for Multiuser VLC Systems

**DOI:** 10.3390/s22072771

**Published:** 2022-04-04

**Authors:** Hafiz M. Asif, Affan Affan, Naser Tarhuni, Kaamran Raahemifar

**Affiliations:** 1 Department of Electrical and Computer Engineering, Sultan Qaboos University, Muscat 123, Oman; tarhuni@squ.edu.om; 2 Department of Electrical and Computer Engineering, University of Louisville, Louisville, KY 40292, USA; affan.affan@louisville.edu; 3 Data Science and Artificial Intelligence Program, College of Information Sciences and Technology (IST), Penn State University, State College, PA 16801, USA; kraahemi@psu.edu; 4 Faculty of Science, School of Optometry and Vision Science, University of Waterloo, 200 University Ave. W, Waterloo, ON N2L 3G1, Canada; 5 Department of Chemical Engineering, Faculty of Engineering, University of Waterloo, 200 University Ave. W, Waterloo, ON N2L 3G1, Canada

**Keywords:** beamforming, deep learning, maximum likelihood, new technologies used in massive MIMO, orbital angular momentum

## Abstract

Due to the growing number of users, power, and spectral effectiveness, most communication systems are complex and difficult to implement on a large scale. Artificial Intelligence (AI) has played an outstanding role in the implementation of theoretical systems in the real world, with less complexity achieving better results. In this direction, we compare the Non-Orthogonal Multiple Access (NOMA) technique for a multiuser Visible Light Communication (VLC) system with Successive Interference Cancellation (SIC) for two types of detectors: (1) the deep learning-based system and (2) the traditional maximum likelihood (ML) decoder-based system. For multiplexing, we compare the variations of novel Orbital Angular Momentum (OAM) multiplexing and Orthogonal Frequency Division Multiplexing (OFDM) with Index Modulation (IM). In this article, we implement OFDM-IM and OAM-IM for four users for the Gaussian fading MIMO Line-of-Sight (LoS) and Non-Line-of-Sight (NLoS) VLC channels. The suggested systems’ bit error rate (BER) performances are compared in simulations for a wide range of Signal-to-Noise Ratios (SNRs), which shows that deep learning-based systems outperform the ML-based system for both users to ensure better decoding at the receiver end, especially at higher SNR values. The detection error is lower in a deep learning-based system at around 20% and around 30% for low SNR and high SNR values, respectively.

## 1. Introduction

### 1.1. Motivation

**Figure 1:**
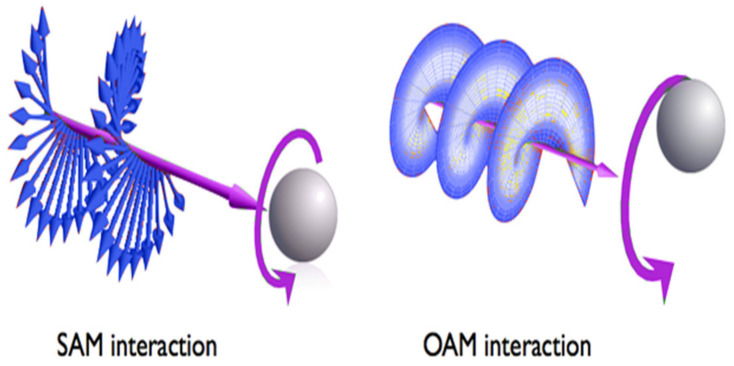
Waveforms of OAM and SAM.

Wireless communication systems such as Visible Light Communication (VLC) have proven to increase spectral efficiency and provide reliable data connections at higher data rates. However, the channel model in VLC can be free space or underwater. The increase in the channel non-linearities that affect the performance of the system requires receiver designs to be more complex [1]. It becomes more challenging when the communication system involves more variables to be estimated. The traditional equalizers and receiver designs can mitigate these effects to some extent. However, Artificial Intelligence (AI) has been proven to be effective in wireless communication systems for modulation detection, jitter mitigation, nonlinearity mitigation, and phase estimation [1,2,3]. Next-generation communication systems are expected to be reliable, robust, spectrally efficient, and fast, which can accommodate the demand of increasing users without sacrificing system performance. Furthermore, the requirements such as environmentally friendly properties, power, and bandwidth efficiency are also top of the chart. Visible Light Communication (VLC) (also known as Li-Fi) is related technology that is a solution to meet these requirements for next-generation communication systems. VLC systems are based on visible light sources, such as Light-Emitting Diodes (LEDs), which are environment friendly and have the spectrum range of 380–780 nm, i.e., a non-interfering range with Radio Frequency (RF)-based communication systems. Despite numerous advantages, VLC systems also face the challenge of handling a large number of users in the context of multiuser communication. It is worth noting, however, that the Orbital Angular Momentum (OAM) of RF waves has been recently the focus of the researchers because of its helical phase wavefront due to successive phase shifts in space [4,5,6,7,8,9]. Similar to the RF waves, the photons of the visible light beam can create a helical phase waveform with a phase front of $\exp\left(il\phi \right)$ for the azimuthal angle, ϕ, per l photon [10]. It is important to mention that OAM is not similar to Spin Angular Momentum (SAM). SAM is associated with circular polarization, and OAM is associated with the spatial distribution of photons as shown in Figure 1. The successive phase shifts create orthogonal OAM modes, which are the key feature of OAM waves, and make multiplexing and demultiplexing the OAM easy with less interference.

One of the advantages of OAM is the all-time availability of traditional resources, such as frequency and time, which can be used to increase spectral efficiency. The OAM wave has multiple orthogonal topological charges and provides a novel technique to dramatically boost spectrum efficiency, and, thus, it is predicted to be a potential multiplexing technique in future wireless communication networks. However, the disadvantage of OAM is the requirement of a high number of LEDs in order to achieve spatial orthogonality, which leads to an increase in the system’s complexity and power consumption. To combat this, the Index Modulation (IM) technique can be integrated with the OAM to increase spectrum efficiency while reducing power consumption at the same time [4,6,7]. IM is a type of digital modulation that uses the ON–OFF mechanism to assign some of the transmitted bits as the indices to other transmitting bits.

In this research, OAM-IM is used for a multiuser communication system using Power-Domain Non-Orthogonal Multiple Access (PD-NOMA). The performance of OAM-IM is compared with that of the Orthogonal Frequency Division Multiplexing (OFDM)-IM-based multiuser communication system. For the receiver design, we primarily used the maximum likelihood (ML) method. However, due to inherent mathematical complexity in ML, which increases with the increase in OAM modes and the number of users [4], an additional deep learning-based approach for Successive Interference Cancellation (SIC) is also used at the receiver end to improve the overall performance of the system. 

### 1.2. Related Work

Recently, various studies have been published in the area of OAM [11,12,13,14]. In [11], the experiments for VLC systems based on OAM are conducted with different Signal-to-Noise Ratios (SNRs), power distribution, etc. In [12], green, red, and blue beams of white light are twisted to generate independent channels, and OAM is used for multiplexing for data transmission. Similarly, in [13], RGB beams are used to increase the degree of freedom for wave generation in the Li-Fi model. In contrast to OAM, due to low Inter-Symbol Interference (ISI) and improved Signal-to-Noise Ratio (SNR). OFDM has been widely used and is well liked in the community of wireless communication [15,16]. A high Peak-to-Average Power Ratio (PAPR) is unfortunately a key OFDM disadvantage; however, many methods have been proposed to address this issue. For instance, in [17], hybrid OFDM-Pulse Width Modulation (OFDM-PWM) for multiuser VLC systems is proposed to convert irrational values into 1s and 0s to drive the LED circuit, which leads to a low PAPR. In [18], ESIM-OFDM with a sigma demodulator is proposed for the VLC system in the experimental setup. In [19], the variants of OFDM are studied for VLC-based systems, and the study shows a comparison of these variants and proposes solutions to different issues. 

**Figure 2:**
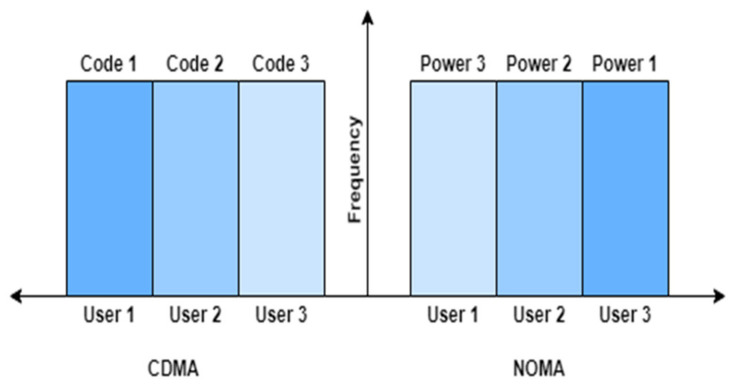
Multiple access using CDMA and NOMA.

To accommodate multiple access in VLC systems, multiple techniques have been proposed in the literature, such as OFDM, Time-Division Multiple Access (TDMA), and Code Division Multiple Access (CDMA) [17,20,21,22,23,24,25]. The source allocation for CDMA and PD-NOMA is shown in Figure 2. In [26], MIMO-OFDM is used for multiuser VLC systems by assigning a single carrier to each user. Similarly, in [11], CDMA is used with OFDM to accommodate multiuser communication instead of the single carrier to each user. These systems have performed very well. However, there are some inherent drawbacks in these techniques. For example, CDMA has degraded performance due to a low SNR among users. Similarly, Orthogonal FDMA (OFDMA) causes spectrum saturation and suffers from a high PAPR. In this research, we utilize PD-NOMA [27,28,29,30,31,32] because NOMA offers better spectrum efficiency, reduced latency, and massive connectivity. In PD-NOMA, the data of the users are suppressed using power difference. Each user’s signal is transmitted with a different and unique power value in the network. At the receiver end, the data of the user with the highest assigned power are recovered by normalizing the received signal with the assigned power factor, while SIC is employed for the retrieval of the data of the remaining users. 

The performance of VLC systems has been widely studied and reported in the literature. In [33], the performance of a multiuser VLC system is studied to reduce SNR fluctuations in order to provide quality services to all users. LED placement is studied in [34] to increase the efficiency of the multiuser VLC system by dividing LEDs into sectors and allocating power concentration. VLC systems have improved over time to meet the requirements of users; however, often, these systems become more complex, which leads to infeasibility for real-world implementation. In this regard, we investigated Artificial Intelligence (AI)-oriented solutions as an alternative to adaptable, feasible, and extendable systems, with the main focus of accommodating a large number of users.

The development of 5G communication systems has already introduced several novel techniques, which highlights the need for techniques that are referred to as 6G or Beyond 5G (B5G) [30]. With the fast processing and increasing number of variables to be optimized in the communication system, implementation complexity increases. Even with a known channel, the accuracy of detection declines with the increase in the number of users. In the case of IM and its variants with OAM and OFDM, detection methods become complex with an increasing number of users and a different activation mode because the signal power of activated modes reduces, and it becomes difficult for traditional methods to accurately detect. In this regard, AI has played an important role due to its capabilities of learning the environment and channel conditions to improve detection accuracy. The system’s performance increases, as the algorithm can learn in contrast to traditional methods that use one-time mathematical function definitions. In [35], VLC-based indoor positioning systems using KNN, ANN, and clustering are discussed, and the effectiveness of these techniques is given. In [36], regression in machine learning is used for indoor positioning using a VLC system. To improve the overall performance of VLC systems, [37] presents machine learning techniques to mitigate non-linear effects. Inspired by the advantages of the low bit-error-rate (BER), low complexity, and high data rate mentioned in the recent literature, we implement OAM-IM and OFDM-IM with a CNN-based approach for SIC to retrieve data for a NOMA-based multiuser VLC system.

In this study, a four-user VLC system is proposed using OAM-IM and OFDM-IM. The BER performance for the Line-of-Sight (LoS) and Non-Line-of-Sight (NLoS) VLC channels with both the maximum likelihood (ML) method and CNN-based detection method is compared. Our primary experiments demonstrate the benefits and drawbacks of OAM-IM and OFDM-IM at various SNR levels. The results show that OAM-IM performs better than OFDM-IM for multiuser VLC systems. In the comparison of CNN- and ML-based systems for NOMA with SIC, the CNN-based system performs better than the ML method. The remainder of this article is as follows: First, we discuss the system model, which includes a detailed mathematical discussion on OFDM-IM, OAM-IM, the VLC channel, NOMA, and CNN-based receiver design. Then, we present the BER results for four users in a MIMO configuration followed by the conclusion.

## 2. Materials and Methods

The theoretical basis of OFDM-IM and OAM-IM is designed in this section. Each technology’s system model is further explained below.

### 2.1. OAM with Index Modulation (IM)

**Figure 3:**
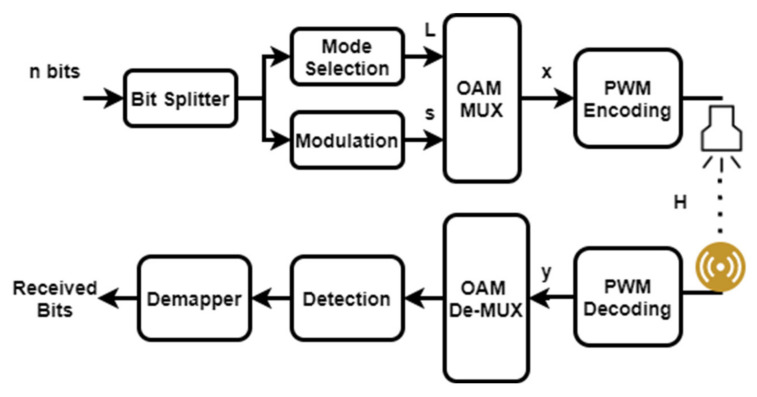
System block diagram of OAM-IM.

This hybrid approach, i.e., combining IM with OAM, reduces power consumption and increases spectral efficiency by utilizing a certain number of OAM modes at a particular time instead of all available modes to store a modulated symbol, and additional information is transmitted by encoding the bits in the form of indices for the active OAM modes. Spectral efficiency depends on the available active modes out of the total active modes, M, and is equal to the number of transmitting LEDs. In the current work, the number of transmitting and receiving resources is similar in a MIMO configuration. $M_{a}\in \left[1,2,\cdots M\right]$ denotes the number of activated modes [4]. The OAM wave is called Mode Division Multiplexing (MDM), which has all the available modes activated, such as $M=M_{a}$. OAM-IM’s spectral efficiency for modulation order N is written as follows:

(1) $$\zeta =M_{a}\log_{2}N+[\log_{2}\left(\begin{array}{c} M\\ M_{a} \end{array}\right)],$$

where [.] is the floor function, used to avoid errors due to the decimal in log operation. The number of bits used for modulation is $M_{a}\log_{2}N$, and $\left|\log_{2}\left(\begin{array}{c} M\\ M_{a} \end{array}\right)\right|$ shows the number of bits used for mode selection by index modulation as shown in Figure 3. The signal applied to the $m^{\mathrm{th}}$ LED is given as follows:

(2) $$x_{m}^{M_{a}}=\frac{1}{\sqrt{N}}p_{M_{a}}s_{M_{a}}\exp\left(-j2\pi \frac{mM_{a}}{M}\right),$$

where $p_{M_{a}}$ is the power assigned to mode M, and $s_{M_{a}}$ is the modulated signal’s $M_{a}^{\mathrm{th}}$ element. The vector s comprises complex constellation symbols, $d\in Se^{j\theta i}$, as well as some zeros; the indices of nonzero items are kept in $L_{i}$, where $L_{i}=\left\{l_{1},l_{2},\cdots l_{M_{a}}\right\}$ is the $i^{\mathrm{th}}$ combination matrix of the activated modes depending on the mode selection bits. It is important to mention that $p_{M_{a}}$ does not refer to the power factor used for NOMA; its sole purpose is to meet the power requirements for transmission.

The signals from different active modes are linearly suppressed together [38]. The transmitted signal can be written as follows:

(3) $$x_{m}=\underset{M_{a}=L_{i}}{\sum }x_{m}^{M_{a}}=\frac{1}{\sqrt{M}}p_{M_{a}}s_{M_{a}}\exp\left(-j2\pi \frac{mM_{a}}{M}\right),$$

(4) pMa=PtδMa1δ1+1δ2+⋯1δM−1,

where $P_{t}$ is the total power available, $\delta_{i}$ is the $i^{\mathrm{th}}$ eigenvalue of VLC channel H, and $p_{M_{a}}$ is the power of each activated OAM mode. The MIMO VLC channel is defined in Section 2.3 Equations (2) and (3) are expressed as a vector as follows:

(5) x=WPs,

where $W\in {\mathbb{C}}^{M\times M}$ refers to the discrete Fourier transform matrix, P represents the power matrix, and the vector $s\in {\mathbb{C}}^{M\times 1}$ contains complex constellation symbols. The indices of complex constellation symbols are kept in $L_{i}$. s can have the following form:

(6) $$s=\left[0\;d_{1}0\;d_{2}\cdots 0\;d_{Na}\right],$$

x is the final output of the modulation block. The signal x is passed by the PWM-encoding block to convert the complex signal into 1s and 0s to drive the LEDs. Then, the signal is transmitted through the VLC channel. The signal y is detected by the photodetector, and the signal is processed through PWM decoding to convert the 1s and 0s in complex symbols for further processing. Therefore, the signal y is passed through the inverse discrete Fourier transform matrix [4,5,38]. The operations at the receiver end can be written as follows:

(7) $$\begin{array}{c} y=Hx+n,\\ W^{T}y=W^{T}Hx+W^{T}n,\\ y_{r}=W^{T}HWPs+W^{T}n,\\ y_{r}=\Lambda Ps+W^{T}n,\\ y_{r}=Gs+W^{T}n, \end{array}$$

where $\Lambda =diag\left(\mathrm{eig}\left(H\right)\right)$ is a defined diagonal matrix, and $y_{r}$ is the received signal. For the ML-based detector, first, the indices of the active modes are identified, and the modulated bits are recovered later. Index detection can be performed using the following expression:

(8) $$\xi_{M_{a}}^{i}=\frac{{\left|y_{r_{m}}\right|}^{2}}{\sigma_{m}^{2}}+\ln\left(\underset{d\in Se^{j\theta i}}{\sum }\exp\left(\frac{-1}{\sigma_{m}^{2}}{\left|y_{r_{M_{a}}}-g_{m}d\right|}^{2}\right)\right)$$

(9) $$\xi^{i}=\underset{M_{a}\in M_{i}}{\sum }\xi_{M_{a}}^{\left[\frac{iM_{a}}{M}\right]}$$

The $i^{\mathrm{th}}$ mode combination can be detected as follows:

(10) $$\hat{ı}=\arg\;\underset{i}{\max}\;\xi^{i}$$

The symbols can be detected as follows:

(11) $${\hat{d}}_{M_{a}}=\mathrm{argmin}\left|y_{r}\left(M_{a}\right)-g\left(M_{a}\right)d\right|,M_{a}\in L_{\hat{ı}}$$

### 2.2. OFDM with Index Modulation (IM)

Similar to OAM-IM, the spectral efficiency of OFDM can be increased by combining OFDM with IM. The coupling of OFDM and IM establishes the frequency domain mode activation style. The main purposes of OFDM-IM are to map the extra information as the number of active OFDM subcarriers and to make the remaining subcarriers zero, which leaves some of the frequency components free, and, thus, they can then be used for the other user. This enhances bandwidth utilization and power consumption, as well as not restricting the number of LEDs. 

**Figure 4:**
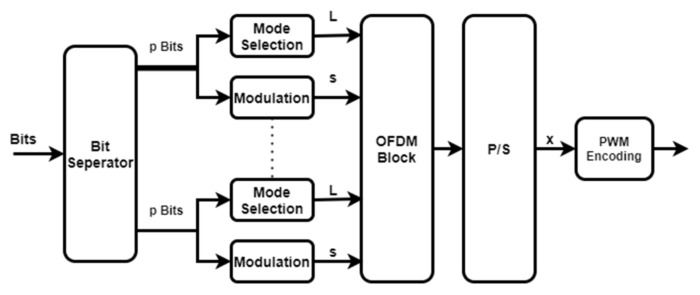
OFDM-IM transmitter system block diagram.

Figure 4 depicts a block diagram of an OFDM-IM-based transmitter. The β bits are broken down into G subblocks. Each subblock for the N number of subcarriers has p bits, p=N/G. Similar to OAM-IM, the p bits are divided into two parts: the first $p_{1}$ bits are used for modulation, and the second $p_{2}$ bits are used for the selection of the indices of the activated subcarriers based on the combination/index bits [5]. $M_{a}$ subcarriers are considered as active subcarriers out of the total M subcarriers. The knowledge of these active subcarriers is kept in $L_{i}=\left\{l_{1},l_{2},\cdots l_{M_{a}}\right\}$, where the elements of $L_{i}$ are the permutations of the activated modes depending on the index bits at a certain time step. OFDM-IM’s spectral efficiency is determined as follows:

(12) $$\beta =GM_{a}\log_{2}N+[\log_{2}\left(\begin{array}{c} M\\ M_{a} \end{array}\right)]G.$$

Only $M_{a}\;$ subcarriers contain data in each subblock G. Each subblock’s output is transformed to serial mode and sent over the VLC channel. The signal x is written as follows:

(13) $$x=\left[\hat{x}\left(1\right),\hat{x}\left(2\right)\cdots \hat{x}\left(N\right)\right],$$

where the components of $\hat{x}$ are made up of nonzero constellation symbols and zero values. The output of each of the G subblocks is written as follows:

(14) $$\hat{x}=\left[s_{1},s_{2},\cdots s_{N_{a}}\right].$$

**Figure 5:**
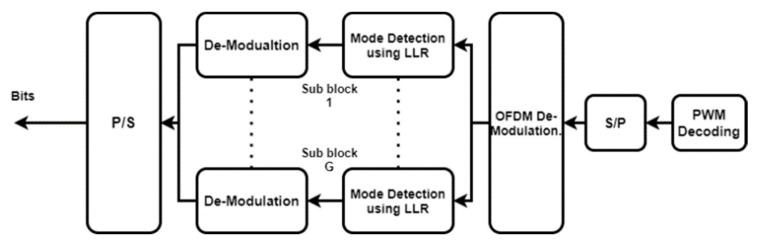
Receiver block diagram of OFDM-IM.

On the receiver side, first, subcarrier indices conveying information bits about activation mode combinations should be recognized [4,5]. The ML-based system is illustrated in Figure 5, and the mathematical working is the same as shown in (8)–(11).

### 2.3. VLC Channel

**Figure 6:**
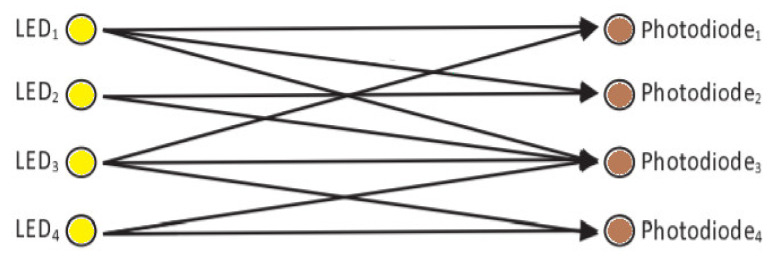
VLC channel MIMO configuration.

Visible light passes through the optical medium for the transmission of data through LEDs to photodetectors. VLC systems experience multiple challenges during the propagation phase, such as shadowing when the illumination location becomes blocked from a certain region. This can also lead to information loss. The LoS and diffused components of propagating visible light form the VLC channel. Figure 6 is an illustration of the VLC channel in MIMO configuration. The main sources of the diffused components in the VLC channel are the walls and other objects in the indoor environment [18]. 

Establishing a point-to-point link is pivotal to effective data communication in VLC systems. This is a challenging task for mobile communication when the receiver is moving. Therefore, it is important to have enough illumination resources that can cover certain indoor areas for uninterrupted data transmission. The VLC channel gain depends on LED illumination intensity, Lambertian order, the size of the photodetector, and several reflective surfaces. The VLC channel gain from the $p^{\mathrm{th}}$ LED to the $q^{\mathrm{th}}$ photodetector is calculated in (15) as follows:

(15) $$h_{p,q}=\left\{\begin{array}{cc} \frac{\rho_{q}A_{q}}{d_{p,q}^{2}}R\left(\phi_{p}\right)cos\left(\varphi_{p,q}\right) & \varphi_{p,q}\leq \psi_{q}\\ 0 & \varphi_{p,q}>\psi_{q} \end{array},\right.$$

where $\varphi_{p,q}$ is the incidence angle from the $p^{\mathrm{th}}$ LED to the $q^{\mathrm{th}}$ photodetector, $\phi_{p}\;$ is the emission angle, $R\left(\phi_{p}\right)$ is the Lambertian radiant intensity, $A_{q}$ is the receiver collection area, $\rho_{q}$ is the responsivity coefficient of the photodetector, $\psi_{q}$ refers to the receiver field of view, and $d_{p,q}$ refers to the distance between the $p^{\mathrm{th}}$ LED and the $q^{\mathrm{th}}$ photodetector. $A_{q}\;$ can be calculated as follows:

(16) $$A_{q}=\frac{\beta^{2}A_{PD,q}}{sin\left(\psi_{q}\right)},$$

where β refers to the refractive index of the photodetector, and $A_{PD,q}$ refers to the area of the $q^{\mathrm{th}}$ photodetector. $R\;\left(\phi_{p}\right)$ can be calculated as follows:

(17) $$R\left(\phi_{p}\right)=\frac{\left(\left(m+1\right)cos^{m}\left(\phi_{p}\right)\right)}{2\pi },$$

where m is the Lambert mode number defining the directivity of the source beam. 

### 2.4. Non-Orthogonal Multiple Access (NOMA)

**Figure 7:**
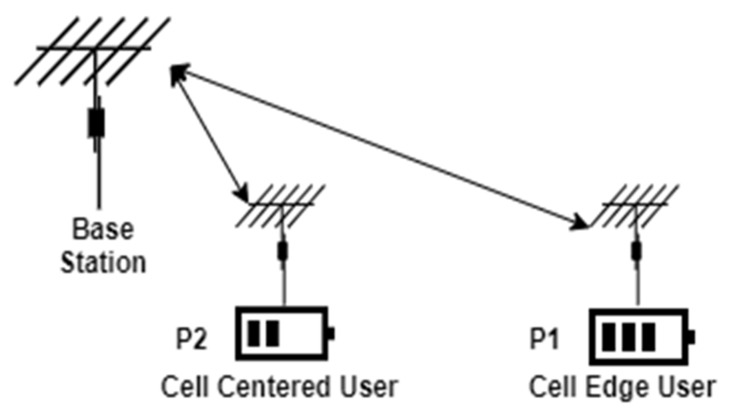
Framework of NOMA.

Power-Domain Non-Orthogonal Multiple Access (NOMA) combines the data of multiple users, and the data are separated using successive interference cancellation (SIC) [27]. In contrast to other multiple access techniques, such as OFDMA and CDMA, NOMA does not use channel spreading or compromise SNR performance. For PD-NOMA, different users use the same resources (frequency and time), which increases the overall system throughput; however, each user has a different power factor. The framework of NOMA is shown in Figure 7 for two users; it can be easily extended for a higher number of users. 

The user at the cell edge is assigned higher power than that of the cell-centered user due to the relative difference in distance from the base station. The received signal can be written as follows [27]:

(18) $$\left[\begin{array}{c} y_{n,1}\\ y_{n,2} \end{array}\right]=\left[\begin{array}{c} H_{n,\;1}\\ H_{n,2} \end{array}\right]\left[p_{1}x_{1}+p_{2}x_{2}\right]+\left[\begin{array}{c} n_{1}\\ n_{2} \end{array}\right],$$

where $y_{n,1}$ and $y_{n,2}$ are the transmitters received at the $n^{\mathrm{th}}$ user; n=1,2. At the receiver end, the data of user 1 are separated and demodulated as follows:

(19) $${\hat{s}}_{1}=\frac{y_{n}}{p_{1}}$$

After the data of user 1 are recovered, the data of user 2 are recovered by mitigating the interference from user 1 using SIC as follows:

(20) $$\hat{y}=y_{n}-p_{1}{\hat{s}}_{1}$$

(21) $${\hat{s}}_{2}=\frac{\;{\hat{y}}_{n}}{p_{2}}$$

## 3. Deep Learning-Based Receiver

**Figure 8:**
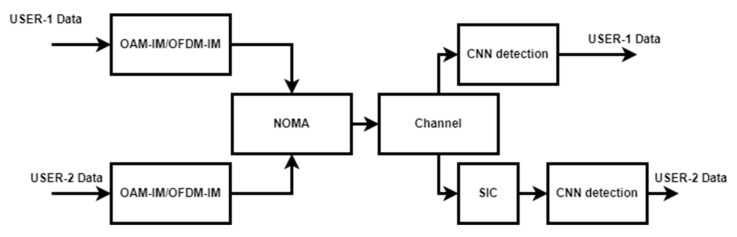
Proposed CNN-based NOMA-SIC multiuser system.

The traditional maximum likelihood decoding scheme becomes practically infeasible and complex as the number of parameters to be detected is increased. Therefore, deep learning-based detection can be investigated as a good alternative detection scheme [38,39]. A Convolutional Neural Network (CNN) with multiple hidden layers is used in this work. The ReLu activation function is used for hidden layers, and a softmax activation function is used for the output layer. Since symbol level detection is considered, we generate training data of constellation symbols of different M-order PSK along with channel information H. The proposed CNN includes three CNN layers followed by the pooling layers and two dense layers, and it ends with the output layer with the softmax activation function. For training, we use the following loss function with $l_{2}$ norm [40]:

(22) $$F\left(\theta \right)=\overset{K}{\underset{k=1}{\sum }}\frac{1}{\left|S_{k}\right|}\underset{s_{k}\in S_{k}}{\sum }{||s_{k}-{\hat{s}}_{k}||}^{2}$$

where k is the constellation symbol, and θ refers to weight and bias. Figure 8 shows the general framework of the multiuser communication system using CNN and NOMA. 

## 4. Results and Discussions

**Figure 9:**
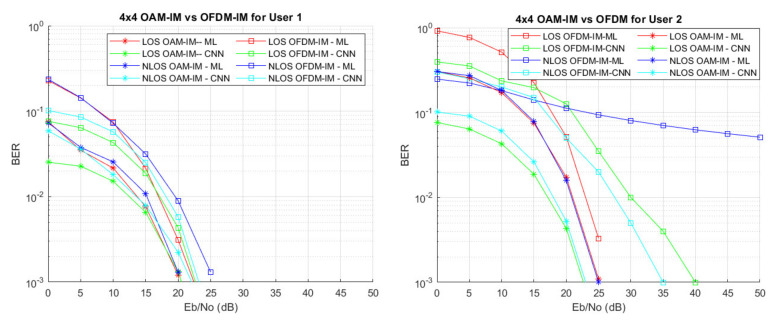
User 1 and user 2 BER of OFDM-IM and OAM-IM for LoS/NLoS VLC channel.

The aforementioned multiplexing techniques, OFDM-IM and OAM-IM are implemented for four users in MIMO configuration in the MATLAB simulation program. For the simulations, the 4-PSK modulator and 4 × 4 MIMO configuration are used. For the sake of comparison, the modulation order is kept the same for the LOS and NLOS VLC channels. The simulation parameters are given in Table 1. It is worth noting that two modes are activated, and the mode combination matrix is shown in (23).

(23) $$L=\left[\begin{array}{cc} 0 & 1\\ 2 & 3\\ 0 & 2\\ 1 & 3 \end{array}\right],$$

where the elements of the matrix, L, show the index of vector x, which has nonzero information. For example, the first row, which contains [0, 1] in the matrix L, shows that the nonzero information will be stored at indices 0 and 1 of the vector. The data of both users are suppressed via NOMA techniques with power ratios of 0.3, 0.4, 0.6, and 0.8 for user 4, user 3, user 2, and user 1, respectively. Figure 9 illustrates the Bit Error Rate (BER) performance of the VLC system based on OAM-IM and OFDM-IM for user l and user 2. It should be noted that both systems are evaluated with an equal number of activated modes. For both users, OAM-IM outperforms OFDM-IM, at least for the low SNR region, irrespective of the detection scheme used. For the high SNR region, the diversity gain becomes relatively more comparable for both schemes. Moreover, the coding gain of OAM-IM is reduced in the high SNR region. Further exploration of OAM-IM based on some parameters such as the rotation angle can lead to better performance in the high SNR region. There are myriad reasons for this enhanced performance of OAM-IM: First, the multiplexing efficient performance of OAM-IM with PD-NOMA is clearly evident. Second, the decoding of OAM-IM is better, which shows it is less effected when transmitted with low transmitted power compared to OFDM-IM symbols. This point is especially valid for the NLOS scenario where the performance of OFDM-IM is notably worse than that of OAM-IM. Third, ML decoding is best suited to OAM-IM in terms of BER performance under PD-NOMA multiplexing. 

As far as the selection of decoding is concerned, CNN-based receiver design performs very well for all ranges of SNR values. The overall dominance of the CNN-based detection method can be observed, as it has low complexity and better results than those of maximum likelihood [18,38]. The BER performance of the ML-based system is good for user 1; however, as the number of users is increased in the system, its performance starts to degrade. It shows that the ML-based method is not as good as CNN due to CNN-based models’ inherent feature of learning the system on the go. The simulation parameters are shown in Table 1 [18]. It should be highlighted that the current work considers a rotation angle of zero for simplicity. However, it is to be noted that the performance of the system can be improved by changing the rotation angles $\left(\theta_{2}\right)$ for different modulation modes for M-PSK.

**Figure 10:**
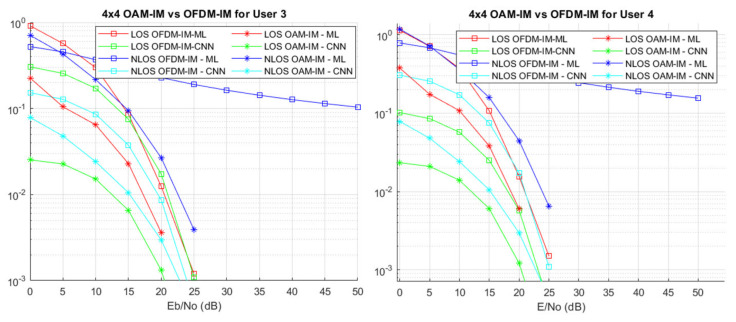
User 3 and user 4 BER of OFDM-IM and OAM-IM for LoS/NLoS VLC channel.

Figure 10 shows very interesting results for user 3 and user 4. Above all, these curves imply that the superior performance of OAM-IM is consistent and is not affected by the increase in the number of users by and large. It can be seen that the difference between OAM-IM and OFDM-IM for a low SNR in Figure 10 is comparatively low; however, as the SNR values are increased, OFDM-IM performance starts to decrease. By contrast, OAM-IM maintains its performance, even for high SNR values. It can be observed that OAM-IM outperforms OFDM-IM for both LoS and NLoS VLC channels. The benefit that OAM-IM has an edge in the sense of spectral efficiency, which is difficult to achieve in OFDM-IM, is also highlighted here. The performance of OFDM-IM with low power degrades when using the maximum likelihood detection method; however, CNN-based receiver design improves the BER for both LoS and NLoS VLC channels for all ranges of SNR values. The results show that the CNN-based receiver design performs better than the ML method. The CNN-based system improves the detection rate by up to 20% and 30% for low and high SNR values, especially for low-power users. The CNN-based VLC system is practically suitable for a higher number of users and activation modes because, due to the increasing number of elements to be optimally detected and the avoidance of interference ML-based systems, system complexity is heightened, which leads to a high computational cost and higher chances of system failure. It is important to mention that, for these results, the channel matrix is known and assumed to be static. 

## 5. Conclusions

We investigated index modulation (IM) with Orbital Angular Momentum (OAM) multiplexing and Orthogonal Frequency Division Multiplexing (OFDM) for the multiuser communication system. To support multiple users, Power Domain Non-Orthogonal Multiple Access (NOMA) was considered. A Successive Interference Cancellation (SIC) mechanism with maximum likelihood (ML) and a CNN-based detection method were utilized at the receiver end to decode the users’ data. The simulation results clearly demonstrated that OAM-IM generally outperforms the OFDM-IM-based VLC system. The use of IM in conjunction with OFDM and OAM showed to enhance system efficiency, at least in terms of the BER parameter. As evident through simulation results, OAM has substantially greater spectral efficiency than OFDM, which was further enhanced by IM. Moreover, the detection error was reduced for both users; however, it was observed to be more significant in users that had a low NOMA power factor. The detection error was further lowered in deep learning-based systems to around 20% and around 30% for low SNR and high SNR regions, respectively. The most significant finding in this study is that AI-based VLC systems can perform better; however, designing an optimal AI network requires effort. Based on the findings of this study, future research will attempt to develop multiuser communication systems using similar methodologies and text in a more realistic environment and explore other AI algorithms, in addition to considering channel estimation algorithms for better suitability of the system. 

## Figures and Tables

**Table 1:** Simulation parameters.

Parameter	Value
Configuration	4 × 4
Average transmission power	1 W
Photodetector’s area	$0.1\;{\mathrm{mm}}^{2}$
Angle of incidence	20°
Distance between LED and photodetector	10 ft
Field of view	120°
Activated modes	2
